# Individual and Societal Economic Burden of Chronic Rhinosinusitis with or Without Nasal Polyps

**DOI:** 10.3390/medsci14010067

**Published:** 2026-02-02

**Authors:** Kjell Erik Julius Håkansson, Steven Arild Wuyts Andersen, Anders Løkke, Ole Hilberg, Rikke Ibsen, Charlotte Suppli Ulrik, Vibeke Backer

**Affiliations:** 1 Department of Respiratory Medicine, Copenhagen University Hospital–Hvidovre, 2650 Hvidovre, Denmark; csulrik@dadlnet.dk; 2 Department of Otorhinolaryngology–Head & Neck Surgery and Audiology, Copenhagen University Hospital–Rigshospitalet, 2100 Copenhagen, Denmark; steven.andersen@regionh.dk (S.A.W.A.); backer@dadlnet.dk (V.B.); 3 Department of Medicine, Little Belt Hospital, 7100 Vejle, Denmark; aloekke@gmail.com (A.L.); ole.hilberg@rsyd.dk (O.H.); 4 Department of Regional Health Research, University of Southern Denmark, 5230 Odense, Denmark; 5 i2Minds, 8000 Aarhus, Denmark; rikke@i2minds.dk; 6 Institute of Clinical Medicine, University of Copenhagen, 2100 Copenhagen, Denmark

**Keywords:** nationwide cohort, pharmacoeconomics, cost of disease, crswnp, crssnp

## Abstract

**Background/Objectives**: Chronic rhinosinusitis (CRS) with nasal polyps (CRSwNP) or without NP (CRSsNP) are common upper airway diseases with major impact on healthcare utilization. Little is known about the overall national financial burden of CRS. We aimed to assess the excess financial burden of CRS from a countrywide perspective. **Methods**: Annual expenditure from healthcare, welfare transfers and foregone income was retrieved from national databases, annualized and compared to matched healthy comparators. **Results**: Of the 303,475 patients included with CRS (mean age 51, 55% female), 18,142 were subclassified as CRSsNP (24%) or CRSwNP (76%). For CRS patients, annual excess healthcare costs were €1315 (1296–1333) compared to comparators. Patients with CRS earned €1356 (1230–1479) less annually compared to comparators. Patients with CRS of working age (18–64 years) had excess welfare transfers (€816 (782–850) compared to comparators, driven by sick leave and disability. Increases in healthcare costs were seen for patients with CRSwNP (€5406 (4860–6012) annually) compared to CRSsNP (€4945 (4293–5696)) driven by increases in CRS-related costs. Total societal burden for the entire cohort was €686,052,898, of which systemic corticosteroid exposure-related conditions represented €20,332,825. Excess welfare transfers represented €174,394,048 annually. **Conclusions**: Chronic rhinosinusitis is associated with a significant financial burden, both in terms of societal healthcare and welfare expenditure and patients’ personal finances due to lost income.

## 1. Introduction

Chronic rhinosinusitis (CRS), either with (CRSwNP) or without (CRSsNP) nasal polyps, is a common disease of the upper airways, with 4–8% of the population estimated to suffer from moderate-to-severe CRS [1]. The hallmark sinonasal inflammation of CRS leads to symptoms such as olfactory dysfunction, nasal congestion and/or discharge, and facial pressure, causing poor quality of life (QoL) and decreased mental health on par with other chronic diseases, such as asthma, cardiovascular disease, and migraine [2]. While effective treatments such as nasal corticosteroids (NCS) and sinus surgery for the approx. 25% of CRS patients with CRSwNP [3] are available, many patients require repeated treatments or courses of systemic corticosteroids (SCS) to reduce their symptom burden [1,4].

The chronicity of CRS leads to repeated healthcare use and thus associated with significant healthcare expenditure, with previous studies showing annual costs of approximately $6 billion in the United States alone [5]. Most studies have focused on healthcare expenditure; however, the indirect costs of CRS due to lost productivity and sick leave are thought to be substantial [6]. Furthermore, previous studies have primarily utilized selected cohorts and/or relied on self-reported data [7,8], making generalizability towards the total financial burden from a societal perspective challenging.

Another aspect of CRS-related morbidity and burden stemming from CRS treatment is the use of SCS. The use of one or two annual courses of SCS in CRSwNP can be considered a “useful addition to nasal corticosteroid treatment in patients with partially or uncontrolled disease” according to the 2020 European Position Paper on Rhinosinusitis and Nasal Polyps [4]. Yet, the use of systemic corticosteroids (SCS) is a known risk factor for conditions such as osteoporosis, type 2 diabetes, and cardiovascular diseases [9]. Evidence from young adults with asthma suggest that lifetime doses > 500 mg of prednisolone are associated with both SCS-related comorbidities and all-cause mortality [10]. As such, a significant fraction of excess healthcare expenditure in CRS can be hypothesized to be due to SCS-related conditions for a subset of patients, yet evidence on the extent is sparse.

The nationwide registries in Denmark allow the capture of individual-level data on healthcare expenditure, welfare transfers, and income and for robust matching to comparators from the background population. In the present study, we aimed to estimate the total cost of CRS and the association between SCS-related conditions and CRS in a nationwide cohort of Danish adults.

## 2. Materials and Methods

### 2.1. Data Sources and Access

The study uses data from the Danish National Patient Registry, the National Laboratory Database, the National Prescription Database, the Health Data Authority, and Statistics Denmark [11]. Data are linked on an individual basis by unique central person registry numbers assigned to all Danish residents. Data are available on application to data sources, as required by Danish law. Approval for data access was provided by Statistics Denmark; no informed consent is required for high-level epidemiologic data in Denmark.

### 2.2. Study Population and Observation Periods

**Figure 1:**
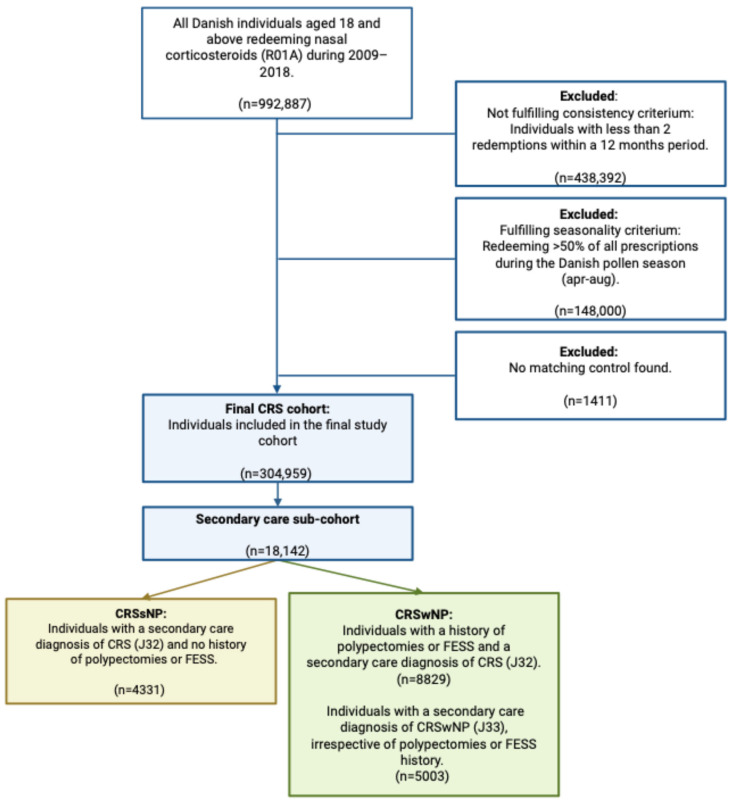
Inclusion flow for a Danish nationwide cohort of patients with chronic rhinosinusitis.

All Danish residents aged above 18 years and fulfilling the inclusion criteria based on previously published definitions [12] during the 2009–2018 period were considered to have CRS and were included in the present cohort (Figure 1). A comparator group of 1:4 matched by age, sex, cohabitation status (married/cohabiting or living alone), and residence at the index date were included. Patients were followed in official databases for individual observation periods of two years post index date, unless censored by dates of emigration or death. 

#### 2.2.1. Inclusion Criteria

(1) Age 18 or above at the index date.
(2) Treatment or diagnosis criterium, either:
  - Treatment: Redemption of physician-prescribed containers of nasal decongestants and/or corticosteroids (ATC-code R01A) within 12 months.
  - Secondary care diagnosis: CRSsNP (ICD-10 J32) or CRSwNP (ICD-10 J33).

#### 2.2.2. Exclusion Criteria

(1) Seasonality exclusion criterium: patients with >50% of R01A redemptions during the Danish pollen season of April through August.
(2) Inconsistent treatment criterium: patients redeeming fewer than 2 prescriptions of NCS within 12 months from their index date.
(3) Individuals without matching comparators.

### 2.3. Definitions

#### 2.3.1. Presence of Nasal Polyps

For patients managed in secondary care, subanalyses were performed according to nasal polyp status. CRS was further subdivided into CRSsNP and CRSwNP based on the additional criteria below. For a complete list of codes used, please see Supplementary Table S1.

CRSwNP was defined as either:

- Secondary care diagnosis code of nasal polyposis (DJ33).
- A history of polypectomy or sinus surgery (e.g., functional endoscopic sinus surgery (FESS)) and a concomitant diagnosis of CRS (DJ32).

CRSsNP was defined as:

- Secondary care diagnosis code of CRS (DJ32). Due to the lack of a separate CRSsNP diagnosis code, patients must not have a history of polypectomies or sinus surgery (e.g., FESS).

#### 2.3.2. Subpopulations

Exploratory analyses based on CRSwNP and need for recurrent surgery were performed, defined as: CRSwNP without history of sinonasal surgery, CRSwNP with history of one sinonasal surgery or CRSwNP with history of more than one sinonasal surgery.

#### 2.3.3. Comorbidity

Comorbidity burden is calculated using Charlson comorbidity index with updated weights by Quan et al. [13,14]. Furthermore, a variable for the presence of known systemic corticosteroid (SCS)-dependent comorbidities not present in the Charlson comorbidity index was constructed to adjust for any SCS exposure-related morbidity driven by non-CRS-related SCS exposure [10], see Supplementary Table S2.

### 2.4. Costs

Costs were stratified according to direct costs (healthcare), welfare transfers and indirect costs (difference in earned income between cases and comparators). Costs, income, and welfare transfers are captured using either diagnosis-related group codes, the Danish ambulant grouping system, the prescription registry or Statistics Denmark’s income and tax databases.

#### 2.4.1. Division of Direct Costs

Direct costs include the patient-facing costs of pharmacy redemptions, healthcare visits in primary sector including general practice and private specialists as well as hospital contacts (outpatient, emergency department, inpatient admissions). Direct costs were further divided into CRS related, SCS related, and other costs. CRS-related costs include costs for all hospital and specialist contacts related to CRS based on diagnosis, procedure/surgery, and imaging codes. Pharmacy redemptions for ATC codes R01A were included in CRS-related costs.

Systemic corticosteroid-related costs were costs related to either hospital contacts or pharmacy redemptions for common pharmacotherapy for frequent SCS-related comorbidities as defined by Skov et al. [10].

A full list of codes used can be found in Supplementary Table S3. Direct costs not fulfilling the above criteria were defined as other direct costs.

#### 2.4.2. Division of Indirect Costs and Welfare Transfers

Indirect costs were defined as foregone income, e.g., the difference in salaried/earned income between cases and comparators.

Welfare transfers were based on student grants, unemployment benefits, social security, age pension, early retirement, disability pension, at-home assisted living and sick leave paid for by the state. For cost prediction, welfare transfers were limited to individuals of working age (18–64) to assess the impact of CRS on the patient population expected to be a part of the workforce.

### 2.5. Statistical Analyses

Cost estimation and tests between groups were estimated using a generalized linear regression model (GLM) with a gamma distribution and a log link. The statistical model is used due to the continuous nature of the outcome, the presence of zeroes in the data as well as the requirement to weight by exposure time, as described by Buntin and Zaslavsky [15], and previously used in other studies [16,17]. The GLM model consisted of a gamma distribution for the expenditures and a log-link function assuming that the logarithm of the expected value of the expenditures could be modelled by a linear combination of parameters on the right-hand side. The model is estimated using maximum likelihood estimation.

Costs were estimated for two years post index and subsequently annualized. Models are adjusted for Charlson comorbidity index, SCS-treated comorbidity and education, in addition to the matching already performed. Prediction estimates were performed for a 50-year-old woman with vocational education, Charlson comorbidity index = 0, and no SCS-dependent comorbidity. Costs are unadjusted for inflation and converted to Euro (€) at a rate of 1 € = 7.45 DKK.

Statistical analyses were performed using SAS version 9.4 (the SAS Institute, Cary, NC, USA) and graphics were generated using ggplot2.

## 3. Results

In a nationwide cohort of all Danish adults, we identified 303,475 individuals (mean age 51 (SD 18), 54.6% female) who fulfilled the inclusion criteria for CRS and were, therefore, included in the present cohort (Figure 1). Statistically significant differences in education level and comorbidity burden were found between patients and their comparators (Table 1).

Overall, 6.0% of patients were managed in secondary care during their follow-up and thus had subclassification data available. Of secondary-care-managed patients, 76.1% were classified as having CRSwNP and 23.9% as CRSsNP. Patients with CRSwNP were more likely to be in active employment and significantly less likely to be female or live in the Capital region compared to patients with CRSsNP (Table 1).

### 3.1. Financial Burden of Chronic Rhinosinusitis

**Figure 2:**
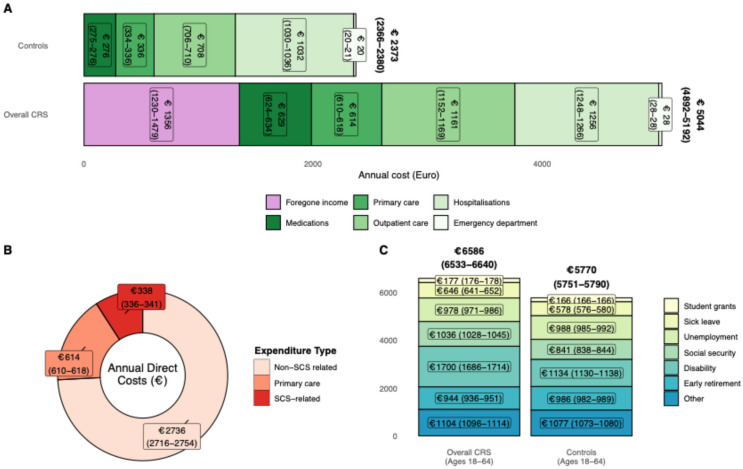
Annual predicted costs (**A**), (**B**) direct costs stratified by expenditure type, and (**C**) direct costs stratified by systemic corticosteroid in relation to patients with chronic rhinosinusitis and age-, sex-, municipality- and cohabitation status-matched comparators.

On an annual basis, the predicted net excess cost of CRS per patient after adjusting for Charlson comorbidity index, systemic corticosteroid-dependent comorbidity and education was estimated as €2671 (95% confidence interval (CI) 2526–2812) per patient, calculated as the differences between healthcare costs and foregone income expenditure between cases and their comparators. Annual excess healthcare costs were €1315 (1296–1333) per patient (Figure 2A). Of the excess healthcare costs, €77 (75–79) were associated with SCS exposure-related diagnoses (Figure 2B).

For patients of working age, annual excess welfare transfers were €816 (782–850), driven by increases in sick leave, social security and disability transfers (Figure 2C).

Pooling of crude, unadjusted costs suggest an annual excess cost of €686,052,898 for patients with CRS, consisting of €511,658,850 stemming from direct costs and foregone income for the overall population and €174,394,048 from excess welfare transfers amongst patients of working age. Excess mean healthcare costs associated with SCS-related diagnoses of €20,332,825 annually (Table 2).

### 3.2. Influence of Polyp Status on Annual Financial Burden

**Figure 3:**
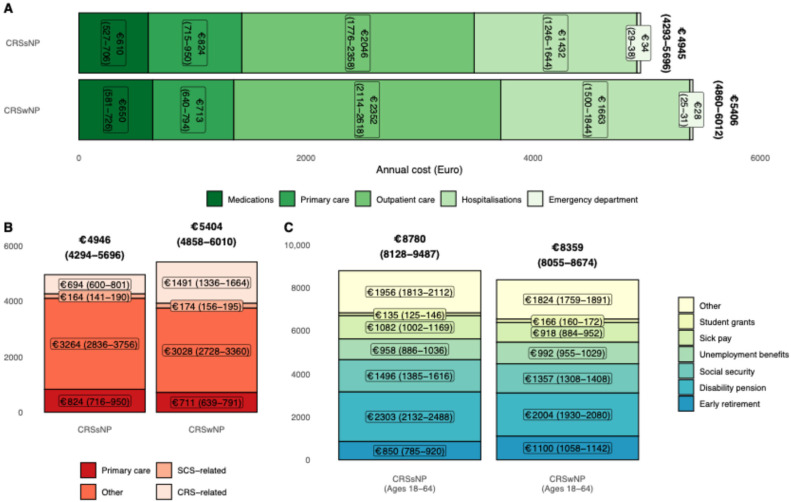
Annual predicted costs (**A**), (**B**) direct costs stratified by disease relationship, and (**C**) welfare transfers (working age only) for patients with chronic rhinosinusitis and without nasal polyps.

Overall annual healthcare costs for CRSsNP and CRSwNP were €4945 (4239–5696) and €5406 (4860–6012), respectively (Figure 3A). When stratified according to CRS relation, CRSwNP had increased CRS-related expenditure (Figure 3B).

For patients of working age, patients with CRSsNP had higher annual welfare transfers compared to CRSwNP at 8780 (8128–9487) and 8359 (8055–8674), respectively. The increase was primarily due to higher use of disability pension transfers (Figure 3C).

### 3.3. Influence of Polyp Burden

For patients with CRSwNP, costs were stratified according to polyp burden assessed as the need for repeated FESS during follow-up. Of the overall CRSwNP population, 83.8% of patients underwent at least one FESS and 11.8% underwent repeated FESS procedures.

**Figure 4:**
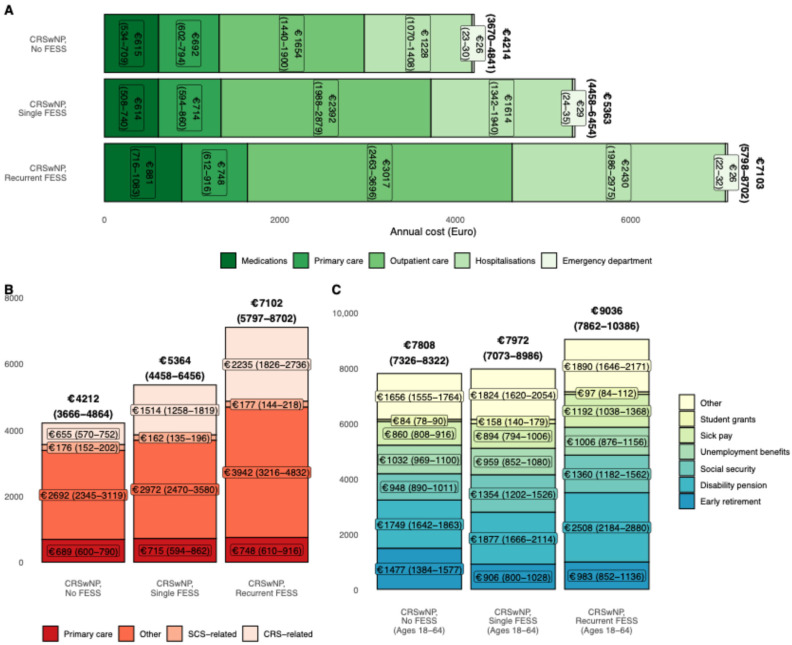
Annual predicted costs (**A**), (**B**) direct costs stratified by disease relation and (**C**) welfare transfers (working age only) with chronic rhinosinusitis with nasal polyps stratified according to sinonasal surgery burden.

Annual predicted healthcare costs for CRSwNP patients without, with a single and with recurrent FESS were €4214 (3670–4841), €5363 (4458–6454) and €7103 (5798–8702), respectively (Figure 4A), driven by increases in costs associated with CRS (Figure 4B).

For CRSwNP patients of working age, those undergoing recurrent FESS during follow-up had increased welfare transfers €9036 (7862–10,386) compared to those with a single (€7072 (7032–8986)) and no FESS (€7808 (7326–8322), driven by higher disability pension transfers (Figure 4C).

## 4. Discussion

Based on a nationwide cohort of 303,475 adults with prescription medication-treated CRS, we estimate the excess annual direct healthcare cost to be €1315 (1296–1333) compared to comparators. We found that patients with CRS had as significant loss of income compared to comparators at €1356 (1230–1479) annually. Finally, patients of working age had increased welfare transfers €816 (782–850) compared to comparators.

This study is amongst the first to document the cost of chronic sinonasal disease in a setting of universal healthcare using individual-level healthcare, welfare and income data for an entire nation, as CRS causes symptoms similar to the common cold, including nasal congestion, albeit all-year compared to other sinonasal diseases such as allergic rhinitis (AR). Indeed, functional limitations and workdays lost are higher in CRS compared to AR [18], and a Swedish cost-of-disease study on AR show reduced per-patient costs compared to the present study at €960 annually per patient [19]. The all-year disease burden and high prevalence of CRS causes a substantial financial burden in Denmark, where crude pooling of the excess costs of CRS estimated a financial burden of €686 million annually.

The annual economic burden of CRS has previously been studied in international studies, with annual costs in the US being estimated to over $30 billion (approx. €28 billion). Translated to per-patient costs, the US financial burden translates to approx. €1900 to 3100 annually [20], in line with our findings. A more recent study, however, found annual costs of up to €29,000 for patients with severe CRSwNP and the need for recurrent surgery [21]. European studies on the costs of CRS are sparse, yet the available literature estimates a per-patient cost of CRS ranging from €1612 to €7160, depending on the presence of polyps, setting, inclusion of indirect costs and the use of excess versus total costs [6,8,22,23].

The presence of nasal polyps, and especially increased polyp burden, is seemingly a driver of financial burden, and particularly prominent for both direct costs and disability pension welfare transfers for patients with CRSwNP compared to CRSsNP. Due to certain procedures being available only in secondary care (e.g., FESS) in Denmark, this creates a referral bias towards more severe cases in the secondary care analyses. However, this incremental increase in costs has previously been demonstrated [5,24]. Previous studies have suggested that recurrent surgeries, SCS use, and increased absenteeism seem to be factors associated with nasal polyps in CRS [5,6]. Of note, CRSwNP patients needing recurrent surgeries demonstrated significant increases in both healthcare-related and welfare-related expenditure compared to those with one or without surgery. While healthcare-related expenditure naturally increases with the number of procedures and has been shown in previous research [21], increases in welfare-related costs suggest that the additional burden of disease and frequent surgery results in both temporary and early workforce withdrawal. We demonstrate a loss in earned wage, arguably a reflection of living with a chronic disease and working reduced hours, lower education attainment and differing choices of careers. Indirect costs such as presenteeism, i.e., lower productivity while present at the workplace, well recognized within CRS, are reduced with some modalities of sinonasal surgery [18,25], and thus the total financial burden of productivity loss is underestimated in the present study. Nonetheless, we found significantly increased utilization of disability pensions, suggesting an increased rate of early, permanent workforce withdrawal. Whether CRS is causative, whether work-related factors have influenced CRS severity [26], or if the increase is due to other reasons remains unknown, yet our findings suggest that patients with severe CRSwNP may be impaired in their daily lives to a level incompatible with a normal working life.

Due to the severely impaired quality of life of many patients living with CRS, and in particular pansinusitis [27], many patients are prescribed repeated courses of SCS and/or recurrent sinus surgeries to try to achieve symptom control [2,3,28]. Evidence for patients’ attitudes towards SCS treatment for CRS is sparse; however, with their frequent use attitudes can be assumed to be similar to asthma where patients are often aware of the risks of repeated SCS treatment, but due to the fast-acting relief provided many accept the long-term tradeoffs [29]. The use of SCS in CRS is supported by evidence of symptomatic and objective relief [30,31,32]. However, in asthma, lifetime exposures to >500 mg have been associated with increased morbidity and mortality [10], suggesting that widespread use of SCS in CRS may provide immediate relief, but recurrent dosing may be detrimental to patients’ long-term health.

The present study is the first, to our knowledge, to investigate the economic consequences of repeated SCS use in CRS. We found that in patients with CRS, common SCS-related comorbidities were associated with an excess financial burden of over €20 million annually and thus in theory preventable using steroid-sparing therapies. Steroid-sparing treatments such as functional endoscopic sinus surgery (FESS) and novel biologics may provide opportunity for reducing the occurrence of SCS-related corticosteroid comorbidities, while reducing CRSwNP-related morbidity. Despite the introduction of corticosteroid-sparing treatments such as FESS and biologics for CRSwNP, there is a continued unmet need in patients with CRSsNP, who, as demonstrated in the present study have a high burden of excess costs suggestive of a high CRS-related morbidity.

It should be mentioned, however, that for steroid-sparing treatments, the cost-effectiveness of FESS versus standard-care nasal corticosteroids and, especially, FESS versus biologics, is hampered by the current high prices of biologic treatments [33,34,35]. Nonetheless, CRSwNP requiring multiple surgeries demonstrated a significant increase in welfare transfer costs driven by disability pension with increasing polyp burden, demonstrating that insufficiently treated CRSwNP leads to increases in societal expenditure beyond just direct healthcare-related costs. As such, a holistic approach beyond direct costs is needed to accurately assess the cost-effectiveness of any treatment.

### Strengths and Limitations

The present study has several strengths, including the use of databases covering all residents of Denmark with a high level of completeness and individual-level administrative and financial data free from recall bias. However, several limitations are worth mentioning. First, the inclusion criteria include non-corticosteroid nasal treatments to allow for non-guideline treated CRS in primary care where diagnosis codes are unavailable for research, which increases the risk of inclusion of, e.g., patients with allergic rhinitis. However, we have allowed for inclusion of non-corticosteroid nasal treatments, as many patients receive non-guideline recommended treatments [36]. Erroneous inclusion of allergic rhinitis, however, is mitigated by the seasonality of redemption and allergen immunotherapy exclusion criteria and subanalyses for patients with CRSsNP/CRSwNP. The final cohort size reflects a Danish CRS prevalence of approx. 6%, in line with other cohorts [37,38]. Second, the differentiation between CRSsNP and CRSwNP is based on diagnosis codes from treating physicians and prior history of nasal surgical procedures. This is due to the lack of a CRSsNP diagnostic code in Denmark, and the clinical knowledge that most patients receive an unspecified CRS diagnosis irrespective of polyp status. Finally, the diagnosis of nasal polyps typically entails nasal endoscopy or a CT. The result of either diagnostic modality is unavailable in Danish registries and the corresponding ICD-10 diagnoses are issued in secondary care (where both modalities are available) and have been used in lieu of gold standard procedure results. Diagnoses, however, as they are diagnosed in secondary care, are primarily issued by specialists in otorhinolaryngology. It should also be mentioned that the data are retrospective in nature and reflect a pre-biologic era of CRS treatment and thus fails to include any additional excess costs incurred by biologic therapies or changes in practice since 2018. Finally, the data are reflective of Danish practices of CRS treatment, and the organization of the Danish welfare and healthcare systems, which might not be applicable in all countries.

## 5. Conclusions

Chronic rhinosinusitis is associated with a significant financial burden, both in terms of healthcare- and welfare-related societal expenditure and affects patients’ personal finances with lost income.

## Figures and Tables

**Table 1:** Baseline characteristics of 303,475 adults with chronic rhinosinusitis.

	Overall CRS N = 303,475	Subcohort: CRSsNP N = 4331	Subcohort: CRSwNP N = 13,811	Comparators N = 1,201,934	*p*-Value	
					CRSsNP versus CRSwNP	CRS versus Comparators
Age (years)	51 (18)	51 (16)	50 (16)	51 (18)	<0.0001	Matched
Female	165,788 (54.6%)	2535 (58.5%)	5122 (37.1%)	657,319 (54.7%)	<0.0001	Matched
Married/cohabiting	200,158 (66.0%)	2916 (67.3%)	9587 (69.4%)	795,843 (66.2%)	0.0096	Matched
Capital region residency	93,117 (30.7%)	1623 (37.5%)	4204 (30.4%)	368,087 (30.6%)	<0.0001	Matched
Education					<0.0001	<0.0001
Primary or secondary only	101,189 (33.3%)	1390 (32.1%)	4474 (32.4%)	428,241(35.6%)		
Vocational	101,390 (33.4%)	1439 (33.2%)	5194 (37.6%)	404,247 (33.6%)		
Higher education (BSc or above)	95,921 (31.6%)	1437 (33.2%)	3939 (28.6%)	334,597 (27.8%)		
Unknown	4975 (1.6%)	65 (1.5%)	204 (1.5%)	34,849 (2.9%)		
Workforce Status					<0.0001	<0.0001
(Self-)Employed	161,146 (53.1%)	2403 (55.5%)	8163 (59.1%)	644,653(53.6%)		
Outside of the workforce	119,575 (39.3%)	1536 (35.4%)	4429 (32.1%)	444,568 (37.0%)		
Under education	16,799 (5.5%)	303 (7.0%)	932 (6.7%)	62,029 (5.2%)		
Other	5955 (2.0%)	89 (2.1%)	287 (2.1%)	50,684 (4.2%)		
Wage income	€21,103 (−6083, 48,289)	€27,026 (−111, 54,163)	€33,783 (5302, 62,264)	22,086 (−4836, 49,008)	<0.0001	0.0727
Charlson score	0 (0, 0)	0 (0, 0)	0 (0, 0)	0 (0)	<0.0001	<0.0001
≥1	14,371 (4.7%)	273 (6.3%)	577 (4.2%)	54,147 (4.5%)	<0.0001	<0.0001
Steroid-dependent comorbidity	16,229 (5.3%)	347 (8.0%)	757 (5.5%)	53,227 (4.4%)	<0.0001	<0.0001

**Table 2:** Annual mean direct costs, welfare transfers and foregone income for 303,475 patients with chronic rhinosinusitis and age-, sex-, municipality- and cohabitation status-matched comparators. All costs in Euro (€).

	Overall CRS N = 303,475	Comparators N = 1,201,934
Direct costs		
Emergency room	30	23
Hospitalizations	1391	1191
Outpatient contacts	1340	888
Primary care	613	335
Medication	655	304
Sum	4028	2739
of which SCS-related	350	283
Foregone income		
Sum	397	0
Excess costs		
Direct costs	1289	
Foregone income	397	
Sum	1686	
Pooled sum *	511,658,850	
Welfare transfers (individuals of working age only **)		
	**Overall CRS** **N = 219,088**	**Comparators** **N = 870,071**
Student grants	594	548
Sick pay	555	496
Unemployment benefits	824	833
Social security	1209	1074
Disability pension	2255	1679
Early retirement	658	704
Other benefits	1183	1148
Sum	7273	6477
Excess costs		
Welfare transfers	796	
Sum	796	
Pooled Sum *	174,394,048	

* Calculated as excess costs from pooled crude mean times the population n. ** Defined as 18–64 years of age.

## Data Availability

The data presented in this study are available on request from the corresponding author due to data are available on application to data sources, as required by Danish law.

## Supplementary Materials

The following supporting information can be downloaded at: https://www.mdpi.com/article/10.3390/medsci14010067/s1, Table S1: Diagnosis, procedure/imaging and billing codes used for inclusion and exclusion. Table S2: Systemic corticosteroid-dependent comorbidities. Table S3: Diagnosis, procedure/imaging and billing codes used for direct costs.

## Abbreviations

The following abbreviations are used in this manuscript:

CRS	Chronic rhinosinusitis
CRSwNP	Chronic rhinosinusitis with nasal polyps
CRSsNP	Chronic rhinosinusitis without nasal polyps
FESS	Functional endoscopic sinus surgery
SCS	Systemic corticosteroids
ATC	Anatomical therapeutic chemical (code)
ICD-10	International classification of diseases, 10th edition (code)
GLM	Generalized linear regression model
CT	Computed tomography
