# Supplementary material for: Individual and Societal Economic Burden of Chronic Rhinosinusitis with or Without Nasal Polyps
Source: Med Sci (Basel). 2026 Feb 2;14(1):67. doi: 10.3390/medsci14010067 (PMC12921935; doi:10.3390/medsci14010067)
Supplement: Supplementary file 1 [file medsci-14-00067-s001.zip › medsci-4106518-supplementary.pdf]

SUPPLEMENTARY MATERIALS

Individual and societal economic burden of chronic rhinosinusitis with or without nasal polyps – a nationwide cohort study

Kjell Erik Julius Håkansson[1,2], Steven Arild Wuyts Andersen[3], Anders Løkke[4,5], Ole Hilberg[4,5], Rikke Ibsen[6], Charlotte Suppli Ulrik[1,7], Vibeke Backer[3]

[1]Department of Respiratory Medicine, Copenhagen University Hospital - Hvidovre, Hvidovre, Denmark

[2]Department of Respiratory Medicine, Copenhagen University Hospital - Bispebjerg, Copenhagen, Denmark

[3]Department of Otorhinolaryngology – Head & Neck Surgery and Audiology, Copenhagen University Hospital - Rigshospitalet, Copenhagen, Denmark

[4]Department of Medicine, Little Belt Hospital, Vejle, Denmark

[5]Department of Regional Health Research, University of Southern Denmark, Odense, Denmark

[6]i2Minds, Aarhus, Denmark

[7]Institute of Clinical Medicine, University of Copenhagen, Copenhagen, Denmark

Corresponding author full contact details:

Name: Kjell E J Håkansson

Address 1: Respiratory Research Unit 237, Hvidovre Hospital, Kettegård Allé 30

City: 2650 Hvidovre, Denmark

Email: kjell.erik.julius.haakansson@regionh.dk

Funding: The present work is funded by an unrestricted research grant from Sanofi.

Word counts: Abstract: ; Main Text: .

Supplementary Table S1
Diagnosis-, procedure/imaging- and billing codes used for inclusion and exclusion.

**Definition**	**Pharmacy redemptions (ATC-code)**	**Diagnostic codes (ICD-10)**	**Procedure codes (SKS-codes)**	**Exclusion criteria**
**CRS**	Two redemptions of R01A within 12 months.			>50% of R01A redemptions during Danish pollen season (April-August)
Subpopulation: CRSwNP		DJ33	1: Polypectomy: KDHB20, KDHB30<br>2: Sinus surgery or polypectomy: KZXX02<br>KDHB40, KDMW00, KDMW99,<br>KDMB10, KDNB20, KDNW99, KDPW00, KDPW10, KDPW99, KEEB00, KEEB99	
Subpopulation: CRSsNP		DJ32		No polypectomy or surgery codes from CRSwNP

Supplementary Table S2
Systemic corticosteroid-dependent comorbidities

Classification	Diagnostic codes (ICD-10)
**Cystic fibrosis**	E84
**Sarcoidosis**	D86
**Primary adrenocortical insufficiency**	E271
**Pneumonitis**	J67-70
**Inflammatory bowel disease**	K50-51
**Inflammatory polyarthropaties**	M05-14
**Systemic connective tissue disorders**	M30-36
**Inflammatory spondylopathies**	M45-46
**Cancer**	C00-99

Supplementary Table S3
Diagnosis-, procedure/imaging- and billing codes used for direct costs.

Classification	Procedure codes (SKS-codes)	Diagnostic codes (ICD-10)	Pharmacy redemptions (ATC-code)	Billing codes (SPEC2-code)
**CRS-related direct costs**				
CRS	**Polypectomies and sinus surgery:** KZXX02, KDHB20, KDHB30, KDHB40, KDMW00, KDMW99, KDMB10, KDNB20, KDNW99, KDPW00, KDPW10, KDPW99, KEEB00, KEEB99 **CRS Imaging:** UXCA40, UXCA45	J33, J32, J31	R01AD01-13	15, 16, 21, 41, 03, 05
**SCS exposure-related direct costs:**				
Osteoporosis		M80-M82	M05	
Fractures		T02, T08, T10, T12, T142, M484, M485, M80, M843, M844, S02 (excl. S025), S12 (excl. S128, S129), S22, S32, S42, S52, S62, S72, S82, S92		
Osteonecrosis		M87		
DM type 2		E11	A10B	
Adrenal insufficiency		E273, E274A, E274C		
Ischemic heart disease		I20-I25	B01, C	
Heart failure		I110; I130; I132; I420; I426; I427; I429; I500; I501; I509		
Depression/anxiety		F32-F33; F40-F41	N06AB	
Peptic Ulcers		K25-28	A02	
Cataract		H25, H26, H28		
Obesity		E66	A08A	
Psychiatric Disease		F31, F2	N05A	
Other/non-disease specific direct costs				
Any costs not assigned by codes above.				
